# Negative Evaluation Bias for Positive Self-Referential Information in Borderline Personality Disorder

**DOI:** 10.1371/journal.pone.0117083

**Published:** 2015-01-22

**Authors:** Dorina Winter, Cornelia Herbert, Katrin Koplin, Christian Schmahl, Martin Bohus, Stefanie Lis

**Affiliations:** 1 Department of Psychosomatic Medicine and Psychotherapy, Central Institute of Mental Health, Medical Faculty Mannheim/Heidelberg University, Mannheim, Germany; 2 Department of Psychiatry, University of Tuebingen, Tuebingen, Germany; 3 Department of Biomedical Resonance Imaging, University of Tuebingen, Tuebingen, Germany; Liaoning Normal University, CHINA

## Abstract

Previous research has suggested that patients meeting criteria for borderline personality disorder (BPD) display altered self-related information processing. However, experimental studies on dysfunctional self-referential information processing in BPD are rare. In this study, BPD patients (N = 30) and healthy control participants (N = 30) judged positive, neutral, and negative words in terms of emotional valence. Referential processing was manipulated by a preceding self-referential pronoun, an other-referential pronoun, or no referential context. Subsequently, patients and participants completed a free recall and recognition task. BPD patients judged positive and neutral words as more negative than healthy control participants when the words had self-reference or no reference. In BPD patients, these biases were significantly correlated with self-reported attributional style, particularly for negative events, but unrelated to measures of depressive mood. However, BPD patients did not differ from healthy control participants in a subsequent free recall task and a recognition task. Our findings point to a negative evaluation bias for positive, self-referential information in BPD. This bias did not affect the storage of information in memory, but may be related to self-attributions of negative events in everyday life in BPD.

## Introduction

During the last decades, many studies have investigated the special meaning of the processing of information related to the self. Self-reference has been shown to influence information processing at an early sensory stage as well as during later stages including cognitive functions such as evaluation and memory [1–4]. For example, healthy subjects describe themselves with more positive traits in comparison to others, which means they have a positive evaluation bias of themselves. They remember self-related information better, and this effect is most pronounced for positive information [2,4–11]. It is assumed that information related to the self is remembered more accurately because it is of higher relevance to the concerning person and therefore results in a deeper processing and encoding [5], while the preference for positive valent information is regarded to serve the protection of a high self-esteem [12,13]. Self-referential processing has been linked to the engagement of distinct cortical midline structures such as the dorso- and ventromedial prefrontal as well as the cingulate cortex [14].

In Borderline Personality Disorder (BPD), self-referential processing appears to be altered. BPD has previously been linked to a negative evaluation bias when BPD patients have to describe themselves: BPD patients have a particularly pejorative view of themselves [15–18], report a high degree of self-criticism [18], and describe their behaviour in social encounters as more quarrelsome and hostile [19]. However, a recent study by Morey et al. [20] suggests that the BPD patients’ distortions in the perception of themselves may also occur as a more advantageous assessment of oneself with regards to the capacity for cooperative relationships or dealing with everyday life in a deliberate and non-impulsive manner.

Recent research [21] suggests that alterations of self-referential processing also affect attributional processes in BPD. In general, attribution is the process by which people explain the causes of events and behaviours. Different theoretical models of attribution processes have been proposed in the past (e. g. [22,23]) and all of these involve the differentiation between internal and external attributions: whether the subject feels personally responsible for events or whether these are caused by the behaviour of others. Together with assumptions on the stability of causes in time and the specificity and valence of events, attributional styles have been linked to mental disorders in the past. For example, subject suffering from a depressive disorder attribute the causes for negative events in an internal, stable, and global style, i.e. they tend to blame themselves for negative events and believe that such events will repeat in the future and in different situations in their lives [24–26]. Yet only one study has addressed attributional style in BPD: Moritz et al. [21] asked BPD patients and healthy control participants to indicate the causes of positive and negative scenarios in the Internal, Personal and Situational Attributions Questionnaire (IPSAQ-R; [24]). The BPD patients attributed both positive as well as negative events to themselves more often than healthy subjects did. This effect was independent of depressive symptoms and suggests that BPD patients tend to overemphasize the importance of their influence on events independently of their emotional valence.

Thus, there is some evidence that self-referential processing is altered in BPD, with BPD patients more often experiencing events as self-related and having a negative evaluation bias of themselves. Since several studies suggest that self-referential processing relies on distinct mental processes with distinct neural correlates, these findings may reveal a specific cognitive impairment in BPD. However, some studies have found that a negative processing bias in BPD is not confined to the evaluation of the own subject, but also affects the evaluation of others: BPD patients evaluated others and their intentions as more brutal and mischievous as well as less trustworthy and approachable [27–30] which suggests that negative evaluations may be a more general alteration of emotional processing in BPD. However, so far, no studies have investigated whether evaluation processes are altered in BPD depending on the chosen reference frame and whether a negative evaluation bias in the evaluation of self-referenced information is linked to alterations in the self-attribution of events depending on their emotional valence.

In this study, we investigated whether the emotional evaluation of and the memory for information is altered in BPD depending on the referential context and the emotional content of the stimuli. We hypothesized that 1) a negative evaluation bias during processing of self-related stimuli in BPD patients will result in the assessment of a higher intensity of the valence and a better memory performance for negative information. For positive information we expect a lower rating of the intensity of the valence accompanied by a reduced memory performance when compared to healthy subjects. We also assumed that 2) BPD patients show a similar bias when information are referenced to others. Finally, we expect that 3) processes of self-attribution are altered in BPD patients and aim to explore whether the patients’ attributional style is linked to alterations in self-referential processing during the judgment of valence. Our findings revealed a negative evaluation bias for positive and neutral stimuli depending on the referential context, alterations in attributional style and a link of particularly the attribution of negative events to the negative evaluation bias in BPD patients.

## Materials and Methods

### Sample

30 female individuals with BPD and 30 female healthy controls (HC) matched according to age and education participated in this study. All participants were informed regarding study procedures and written informed consent was obtained. The study followed the Declaration of Helsinki. The Research Ethics Board II of the University of Heidelberg, Germany, approved the study, including the study population and the consent procedure.

General exclusion criteria were traumatic brain injuries, current lifetime schizophrenia or bipolar I disorder, mental or developmental disorders, substance dependency during the last year, and substance abuse in the last two months. BPD patients had to meet DSM-IV criteria and be without or on the same, stable psychotropic medication for at least two weeks. HC had no acute or lifetime mental illness and no psychotropic medication.

Clinical diagnoses were assessed by trained diagnosticians using the Structured Clinical Interview for DSM-IV Axis I Disorders (SCID-I, [31]) and the borderline section of the International Personality Disorder Examination (IPDE, [32]). Self-report measures included questionnaires on borderline symptom severity [Borderline Symptom List short version (BSL-23, [33])], depressive symptom severity [Beck Depression Inventory (BDI; [34])], and attributional style [German version of the Attributional Style Questionnaire (ASF-E, [35])]. The latter comprises subscales that differentiate internal, stable and global attributions of negative and positive events.

Demographic data and clinical characteristics are reported in Table 1. While age and educational level did not differ between BPD patients and healthy controls, BPD patients scored higher in every measure of symptom severity. 19 (63.3%) of the BPD patients were free of psychotropic medication, 6 (20%) received an atypical antipsychotic, 5 (16.7%) selective serotonin reuptake inhibitors, 4 (13.3%) serotonin-norepinephrine reuptake inhibitors, and 1 (3.3%) each monoamine oxidase inhibitors, tetracyclic antidepressants, neuroleptic medication, and methylphenidate.

**Table 1:** Demographic and clinical variables in healthy control participants (HC) and patients with Borderline Personality Disorder (BPD).

	**HC (n = 30)**		**BPD (n = 30)**		**Statistics**	
	AM	SD (±)	AM	SD (±)	t*	p
**Age-** years	26.13	7.29	26.10	4.76	0.21	.983
**Years of education**, *n (%)*						
9 years	0	(0)	4	(13.33)	U = 409	.492
10 years	13	(43.33)	10	(33.33)	Z = −0.69	
13 years	17	(46.67)	16	(53.33)		
**BDI**-total score	2.50	3.07	28.79	9.56	−14.33	<.001
**BSL-23**—mean score	0.10	0.15	2.42	0.71	−17.55	<.001
**ASF-E**						
negative events						
— internality^a^	62.44	13.37	88.09	17.14	−6.13	<.001
— stability^b^	56.04	14.60	80.92	16.96	−5.78	<.001
— globality^a^	49.22	16.17	85.54	17.16	−8.00	<.001
positive events						
— internality^b^	79.11	12.62	60.85	17.90	4.36	<.001
— stability^b^	76.50	9.88	68.30	12.52	2.67	.010
— globality^b^	77.35	16.11	65.19	16.45	2.77	.008
**Co-morbidities**, *n (%)*						
major depressive disorder			2	(6.67)		
dysthymia			2	(6.67)		
panic disorder with agoraphobia			2	(6.67)		
social phobia			8	(26.67)		
specific phobia			2	(6.67)		
obsessive compulsive disorder			2	(6.67)		
posttraumatic stress disorder			17	(56.67)		
somatization disorder			1	(3.33)		
unspecific somatoform disorder			2	(6.67)		
bulimia nervosa			2	(6.67)		
binge eating disorder			5	(16.67)		
dissociative convulsions			1	(3.33)		

Note: ASF-E = Attributional Style Questionnaire for Adults; BPD = borderline personality disorder; BSL-23 = Borderline Symptom List-23; BDI = Beck Depression Inventory; HC = healthy control participants; t-Test performed at a significance level of p<.05.

* if not otherwise specified

^a^ missing data of 3 HC and 2 BPD

^b^ missing data of 3 HC and 3 BPD

### Experimental tasks

All subjects performed a valence judgment task adapted from Herbert et al. [4]. During this task, positive and negative valent as well as emotionally neutral nouns were presented with three different referential contexts. Subjects had to rate the emotional valence of the nouns using a 9-point-scale ranging from ‘negative’ to ‘positive’. The valence judgment task was followed by an incidental free recall and a recognition task.

In the valence judgment task, stimuli were nouns naming objects (e.g. waste, bottle, palace), events (e.g. crime, conference, success), or abstract terms (e.g. disadvantage, example, talent) and were selected from a word data base from Herbert et al. [4]. With help of arousal and valence assessments (7 point Likert scale) provided in the database, we selected 180 stimuli to form 3 stimulus classes: 60 positive and 60 negative words with high positive or negative valence and high arousal (valence: positive 1.91 ± 0.30, negative −1.70 ± 0.38, arousal: positive 2.98 ± 0.47, negative 3.42 ± 0.47) and 60 neutral words with low arousal (2.06 ± 0.26) and of medium valence (0.24 ± 0.34). For each of the three valence conditions, the 60 words were split into 3 subsets with 20 words each which were comparable with regards to word length and which were used in the three reference conditions. The assignment of noun subsets to reference conditions was balanced across subjects (for further information on the used stimulus material, please contact the corresponding author).

We varied the reference context by presenting a) a first person singular pronoun for self-reference (e.g. “my”); b) an acquaintance name in genitive case (e.g. “Maria’s”); and c) a definitive article as control condition (“the”). The acquaintance name was determined by asking the participants to pick the name of a female person who was neither positively nor negatively connoted. Participants indicated the person’s approximate age and rated the chosen person regarding their type of relationship and closeness (Unidimensional Relationship Closeness Scale, [36]). Age, relationship type, and closeness ratings did not differ between BPD patients and healthy controls.

Each trial was started by the presentation of the pronoun for 1000ms. This was followed by the presentation of a noun which was ended by the rating response of the participants. A fixation cross was presented during the inter-stimulus interval (ISI, mean duration: 1000 ms, range 600–1400 ms). ISIs were adjusted for reaction times by adding the difference between 3000 ms and the reaction time of the last rating. Stimuli were presented in a pseudorandom order. All stimuli were presented on a 15 inch computer screen, in white letters on a black background, centred on the computer monitor. The software Presentation® (http://nbs.neurobs.com) was used for stimulus presentations.

Immediately following the valence judgment task, participants were asked to write down as many of the nouns presented during the valence judgment task as they could remember. This free recall task was followed by an incidental recognition task: noun stimuli used in the valence judgment task were presented together with 180 nouns which had not been part of the stimulus sets. Participants had to indicate by pressing a button whether or not they recognized nouns from the valence judgment task. The previously presented words and the new words were matched for word-length, valence, and arousal. Stimuli were presented in random order.

### Statistical analysis

Dependent variables were mean valence ratings (valence judgment task), percent words correctly recalled (recall tasks), and percent correct responses (recognition task). For the recall task, absolute frequencies of correctly recalled words were transformed to percentage of all recalled words per condition, after proving that both groups display equal recall performance with the Mann-Whitney-U-Test for independent samples. Statistical analyses were done with repeated-measure analyses of variance (ANOVA) with group (HC, BPD) as between-subject factor and valence (negative, neutral, positive) and reference (article, self-reference, other-reference) as within-subject factors. Statistical analyses of the attributional style measured by the ASF-E was done by 2x2x3-ANOVA with the independent factor group and the repeated measurement factors ‘valence’ (positive vs. negative events) and attributional dimension (‘internality’ vs ‘stability’ vs ‘globality’). Degrees of freedom in the ANOVAs were corrected according to Greenhouse-Geisser correction if appropriate. Post-hoc comparisons were done with t-Tests (Bonferroni-corrected for multiple comparisons). All analyses were performed with IBM SPSS Statistics 20 (IBM, USA).

To explore whether alterations in valence ratings seen in BPD were related to BPD symptom severity, depressive mood, or attributional style, we calculated Pearson’s correlation coefficient between these and the BSL scores, BDI scores, and the ASF-E subscale scores.

## Results

### Valence judgment task

**Figure 1:**
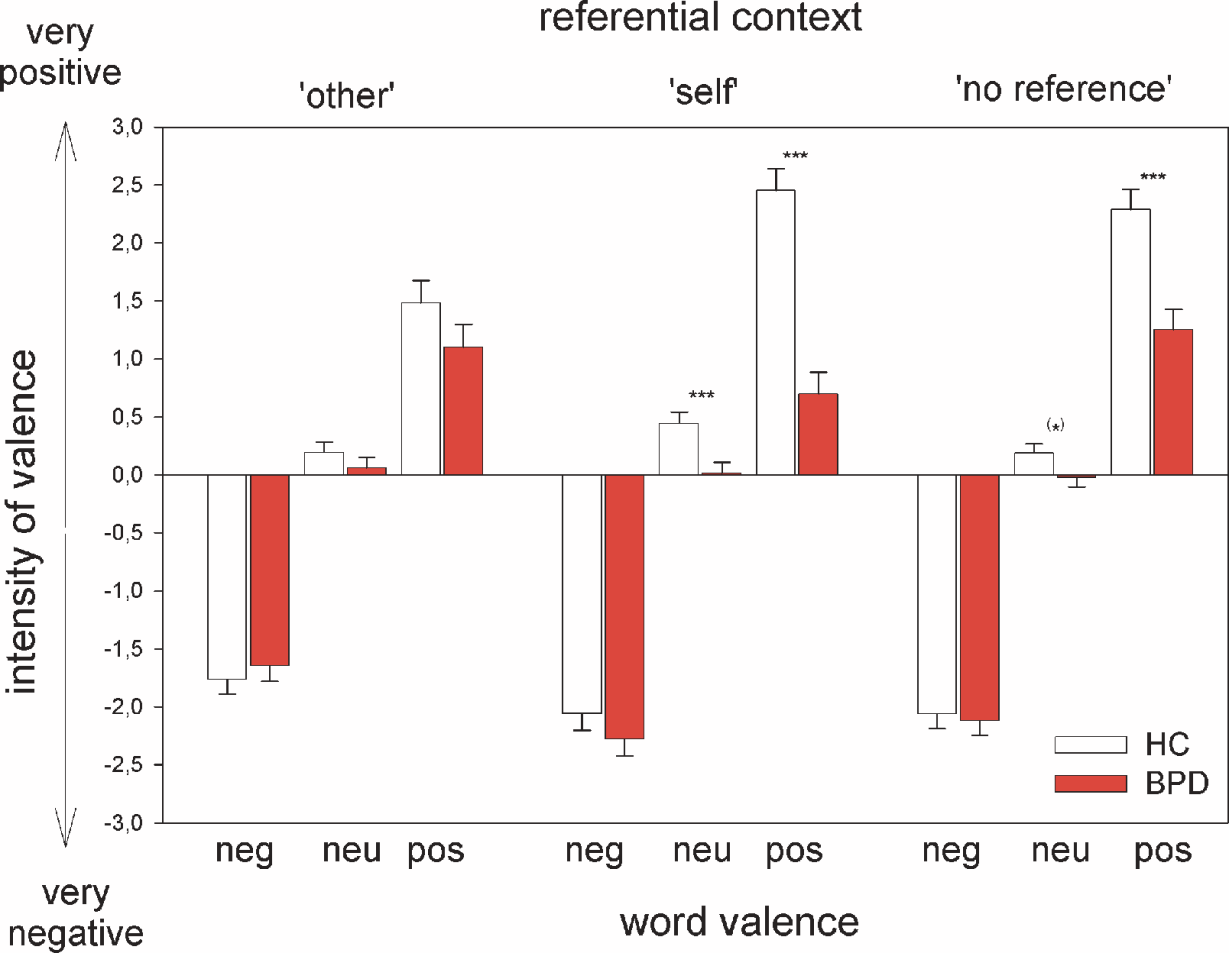
Word appraisal depending on referential context and word valence. Valence ratings of nouns depending on valence and referential context for healthy controls (HC) and patients with Borderline Personality Disorder (BPD). (*) p<.10, p**<.01, *** p<.001.

Means and standard deviations (SD) are summarized in Table 2 and in Fig. 1. Repeated measures ANOVA results are reported in Table 3. The three way interaction Group x Valence x Reference was significant (F_2,139_ = 5.67, p = 0.002, η² = .09): BPD patients rated neutral and positive words less positively than HC if they referred to themselves or had no reference (trend for neutral words). That was not the case for the rating of negative words. No differences between groups were found in the ‘other’-reference condition. Two-Way-ANOVAS were significant, but interpretability was limited due to the higher order interaction (see Table 3). All effects were replicated when computing a comparable repeated measures ANCOVA with medication status (psychotropic medication or not) as covariate (three way interaction: F_2,136_ = 3.49, p = 0.026, η² = .06), even though no significant group difference was observed in the post hoc test for neutral words without reference.

**Table 2:** Rating scores in the word valence judgment task and performance in the memory tasks in healthy control participants (HC) and patients with Borderline Personality Disorder (BPD).

	HC (n = 30)						BPD (n = 30)					
	no reference		self-reference		other-reference		no reference		self-reference		other-reference	
	AM	SD (±)	AM	SD (±)	AM	SD (±)	AM	SD (±)	AM	SD (±)	AM	SD (±)
**Valence judgment task**												
negative words	−2.06	0.73	−2.06	0.86	−1.76	0.72	−2.12	0.65	−2.28	0.73	−1.65	0.72
neutral words	0.19	0.33	0.44	0.34	0.19	0.41	−0.02	0.54	0.02	0.67	0.06	0.56
positive words	2.29	0.77	2.45	0.77	1.48	0.95	1.25	1.10	0.70	1.21	1.10	1.17
**Free recall—**% correct^a^												
negative words	9.52	7.69	10.67	8.89	10.42	8.06	10.00	9.24	13.13	11.64	8.87	7.81
neutral words	6.94	6.99	9.59	7.14	10.77	9.21	6.23	6.12	10.87	9.65	8.47	7.00
positive words	12.63	7.74	16.35	9.90	13.35	8.62	13.50	11.83	16.67	10.78	12.63	10.04
**Recognition task—**% correct												
negative words	70.50	15.99	71.67	15.39	68.33	10.85	74.67	18.89	73.17	18.78	71.83	17.14
neutral words	73.17	17.54	70.50	19.27	67.83	18.08	77.17	14.00	74.50	17.44	75.50	16.73
positive words	77.33	17.51	78.83	14.48	78.33	16.88	79.33	15.85	77.17	20.50	78.33	15.52

^a^ of all correctly recalled words

**Table 3:** Results of the repeated measures ANOVA of word valence ratings with group (healthy controls, Borderline Personality Disorder patients), valence (negative, neutral, positive) and reference (article, self-reference, other-reference).

**Valence judgment task: repeated measures ANOVA of word ratings**			
	*F*	*p*	*η* ^2^
Main effect group	24.71	<0.001	0.90
Main effect valence	416.14	<0.001	0.88
Main effect reference	0.29	0.690	0.01
Interaction group x valence	9.23	0.002	0.14
Interaction group x reference	18.68	<0.001	0.24
Interaction valence x reference	14.81	<0.001	0.20
Interaction group x valence x reference	5.67	0.002	0.09

## Recall task

BPD patients did not differ from HC in overall recall performance (HC AM = 16.90 ± 10.03 SD; BPD AM = 1 6.17 ± 9.30 SD; U = 430.50, Z = −.29, p = .773). The factors valence and reference influenced recall performance (main effect valence F_2,116_ = 16.11, p<0.001, η² = 0.22, main effect reference F_2,116_ = 4.67, p = 0.011, η² = 0.08), however, these effects were not modulated by the factor group: positive words were recalled better than neutral and negative words and recall was better for words with self-reference than words with no reference, but not statistically distinguishable from recall of words with other-reference. All effects were replicated when computing a comparable repeated measures ANCOVA with medication status as covariate (main effect valence F_2,114_ = 9.55, p<0.001, η² = 0.14, main effect reference F_2,114_ = 5.73, p = 0.004, η² = 0.09).

### Recognition task

Recognition performance analysis revealed a significant valence effect (F_1,100_ = 13.667, p<.001, η² = .191): positive words were remembered better than neutral and negative words. There were neither significant main effects for reference or group nor interactions between these factors (see Table 3). A repeated measures ANCOVA with medication status as covariate revealed similar results (main effect valence F_2,114_ = 10.767, p<0.001, η² = 0.16).

### Attributional style

**Figure 2:**
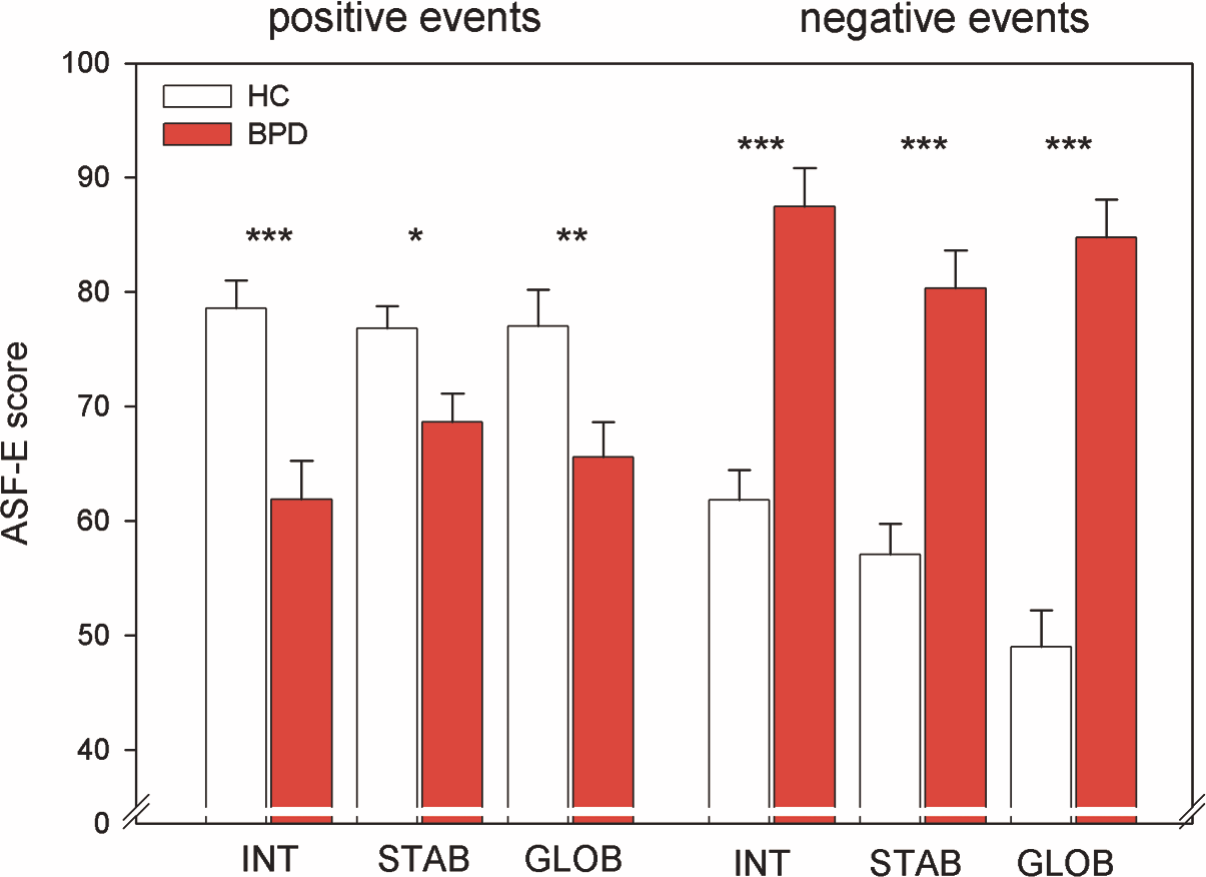
Altered attributional style in Borderline Personality Disorder. ASF-E results on internality (INT), stability (STAB) and globality (GLOB) of attributions for positive and negative events in healthy controls (HC) and patients with Borderline Personality Disorder (BPD). (*) p<.10, p**<.01, *** p<.001.

Statistical analysis revealed differences between BPD patients and HCs modulated by both the valence of the events as well as the attributional dimension (3-way interaction (F_1,94_ = 6.556, p = .003, η² = 0.108). BPD patients assessed the causes for negative events as more internal, stable, and global and for positive events as less internal, stable, and global in comparison to the healthy controls. While for positive events the differences between groups across attributional dimensions were of similar size, group differences were most pronounced for the attributional dimension ‘globality’ when the causes of negative events had to be evaluated. See Fig. 2.

### Exploratory correlational analysis

The reduced positive ratings which were observed in the BPD groups in relation to the other-referential processing condition may be related to BPD symptoms, depressive symptoms, or attributional style. For explorative purposes, we calculated correlations of the difference between the ratings of other- vs. self-referential stimuli separately for positive and neutral nouns with BSL, BDI and ASF-E subscale scores.

**Figure 3:**
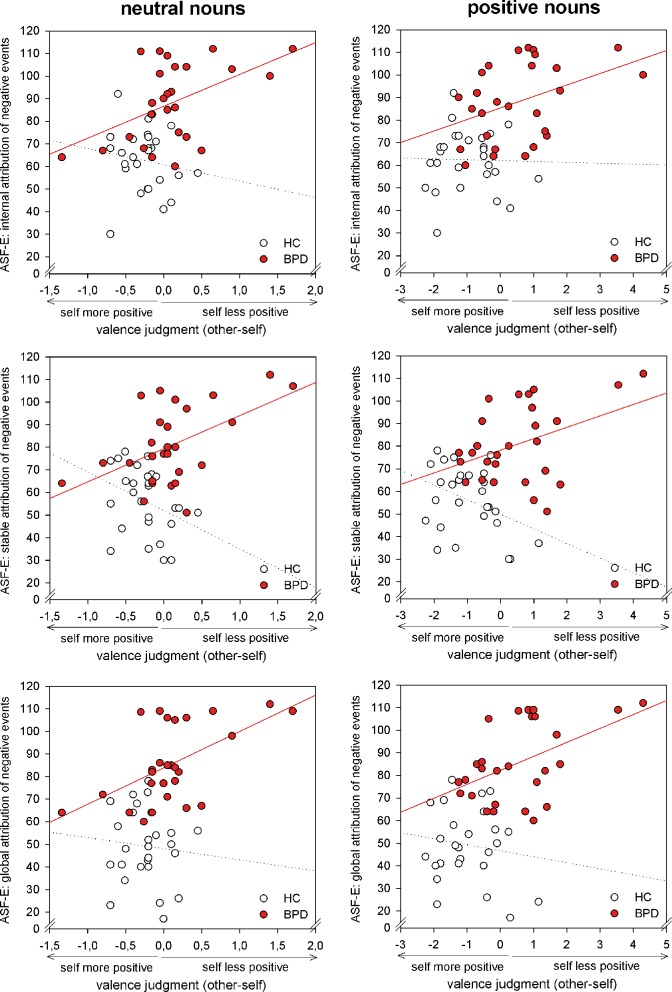
Correlations between self-referential appraisal bias and attributional style. Healthy controls (HC) and patients with Borderline Personality Disorder (BPD) differ in correlations of valence ratings referred to the self in comparison to others and internal, stable and global attributions of negative events for positive and neutral nouns.

Our analyses revealed no correlation of valence ratings with BSL or BDI scores (all p>.1). However, valence ratings were differentially linked to the attributional style of BPD patients and healthy controls (see Table 4): the more pronounced a negative bias during the evaluation of positive and neutral words in relation to the participant herself as compared to the evaluation of information linked to others, the more internal, stable and global the attributional style for particularly negative events in BPD. This covariation did not exist for healthy subjects. This differential linkage of evaluation processes and attributional style between groups was confirmed by significant differences in Pearson’s r between groups (except for the internal attribution of positive events for which a comparison of the two correlation coefficients did not reach statistical significance, see Table 4). In BPD patients, the attribution of positive events was less consistently linked to the self-reference related valence judgments: the more pronounced a negative bias during the evaluation of positive and neutral words in relation to the participant herself in comparison to the evaluation of information linked to others, the less global the attributional style for particularly positive events in BPD. Although no comparable covariation could be observed in the HCs, difference in Pearson’s r between groups could not be confirmed statistically. Statistical analyses revealed a group difference in Pearson’s r for the internal attribution of positive events. However, within groups, correlation was only a statistical trend (see Fig. 3).

**Table 4:** Pearson correlation between alterations in self-referential processing in the valence judgment task and attributional style in healthy control participants (HC) and patients with Borderline Personality Disorder (BPD).

	**HC (n = 30)**				**BPD (n = 30)**							
	**positive words: other-self reference**		**neutral words: other—self reference**		**positive words: other-self reference**		**neutral words: other—self reference**		**Group differences in correlations (BPD-HC, two-tailed)**			
									**positive words**		**neutral words**	
	r	p	r	p	r	p	r	p	Z	p	Z	p
**BDI–** total score	0.12	.526	0.11	.549	−0.01	.947	−0.04	.847	*−0.50*	*.621*	*−0.56*	*.578*
**BSL-23—** mean score	0.02	.911	0.16	.405	0.28	.128	0.23	.231	1.00	.319	0.26	.798
**ASF-E**												
negative events												
− internality^a^	−0.02	.900	−0.16	.427	0.40	.040	0.49	.009	1.56	.120	2.45	.014
− stability^b^	−0.38	.050	−0.34	.080	0.40	.039	0.51	.006	2.85	.004	3.21	.001
− globality^a^	−0.14	.479	−0.09	.659	0.48	.011	0.56	.002	2.34	.019	2.53	.011
positive events												
− internality^b^	0.24	.234	−0.11	.600	−0.35	.070	−0.32	.100	−2.09	.036	−0.77	.441
− stability^b^	−0.04	.856	−0.22	.282	0.02	.914	0.18	.378	0.20	.842	1.38	.168
− globality^b^	−0.21	.291	−0.26	.187	−0.57	.002	−0.47	.011	−1.48	.139	−0.85	.395

Note: ASF-E = Attributional Style Questionnaire for Adults; BDI = Beck Depression Inventory; BPD = borderline personality disorder; BSL-23 = Borderline Symptom List-23; HC = healthy control participants;

^a^ missing data of 3 HC and 2 BPD, ^b^ missing data of 3 HC and 3 BPD

## Discussion

We investigated whether the valence judgment of and the memory for information is altered in BPD depending on the referential context and the emotional content of the stimuli. We also assessed whether such alterations are linked to the attributional style, i.e. to the causes subjects chose to explain positive and negative events. Our findings revealed a strong effect of the chosen reference frame: BPD patients assessed particularly positive and neutral stimuli related to themselves as less positive compared to healthy controls. This devaluation was linked to alterations in attributional style, i.e. to the assignment of more internal, stable and global causes for particularly negative events. In contrast, no alterations in memory processes in BPD patients could be confirmed.

Results of the valence judgment task support the notion of general alterations in self-referential processing in BPD. Indeed, our findings suggest a negative evaluation bias when patients have to evaluate the valence of stimuli being related to themselves. However, this negative bias was not caused by a higher intensity in the evaluation of negative stimuli, but by a lack of a positive bias during the evaluation of positive and neutral stimuli which typically characterises self-referential processing in healthy controls. This finding is in line with findings of Unoka et al. [37] who investigated whether the valence of social signals influences the recognition of emotions in BPD. They showed that reduced performance in a “Reading the Mind in the Eyes” task is caused by deficits in the recognition of positive and neutral stimuli, while no differences in perceiving negative emotions based on the eye region of a face were found in BPD. Similarly, a reduced feeling of belonging was observed during social situations of inclusion in BPD, suggesting that positive social encounters are experienced as being less positive compared to healthy individuals [38].

BPD patients are known to have low self-esteem [15] and a shame-prone self-concept [16,17] with high levels of self-criticism and feeling of inferiority [18]. In subjects high in self-esteem, the experience of positive self-related stimuli is assumed to serve to maintain a high self-esteem. However, in subjects with low self-esteem such as BPD patients, positive stimuli may invoke feelings of shame [12,13] that may result in a devaluation of positive value. Therefore, positive self-related information may not induce the same positive representations in BPD as in healthy control participants. This is in accordance with the theoretical view of Bender and Skodol [39], who assumed that the central problem of BPD patients is the decreased ability to maintain and use kind and integrated internal images of the self, which Bender and Skodol postulate results in interpersonal problems.

To test for the specificity of alterations in self-referential processing in BPD, we used two additional experimental conditions of which one referred stimuli to another individual and the other gave no explicit reference at all. Our findings clearly indicate that evaluating the valence of a stimulus in relation to another subject is not altered in BPD. However, we found a similar effect as that observed for self-referential processing when no explicit reference frame was present. These findings suggest that patients tend to refer information to themselves when no explicit reference context is set. This interpretation is in line with findings from van den Heuvel, Derksen et al. [40] that point to heightened levels of overgeneralization of negative and positive events in relation to the self and particularly across situations in BPD. However, our data contradict previous studies that found that BPD patients tend to interpret the features and intentions of others as more negative [27–30]. These discrepant findings may be explained by differences in the cognitive evaluation processes that have been induced by the different experimental approaches. Previous studies may have induced implicitly a self-referential perspective in that e.g. the evaluation of the trustworthiness of a specific individual may be evaluated in relation to the own person; i.e. in previous tasks other-related information might have been of relevance for the self. It would be useful if future studies investigate whether a negative bias in the evaluation of the personality traits of others depends on whether these traits refer to social attributes of an individual such as ‘hostile’ and ‘friendly’ or describe features that are less essential during interactions with others such as ‘intelligent’ and ‘lazy’. Such studies would clarify whether the chosen stimulus material of the present study − such as objects, events, and abstract concepts instead of adjectives describing personality features−contributed to our findings. Future studies have to manipulate semantics of the word material to disentangle possible effects of these factors.

Although BPD patients differed from healthy controls in the evaluations of emotional, self-referenced stimuli, our data revealed no effects of this altered processing for the storage of information in memory. This held true for both the recall as well as the recognition task and suggests that the differences in evaluation of information have not affected the depth of processing of information. Our findings are in line with literature suggesting that BPD patients do not show a stronger memory bias for emotional information (for reviews see [41,42]). However, it has to be mentioned that in the present study, healthy controls did not show a self-referential bias for positive information as was expected based on previous findings [4,8,9]. Still, all participants remembered self-referential information better than that referring to another person or without referential context. Possible explanations such as design features regarding the duration of the retention interval or the combination of presenting stimuli combined with a valence evaluation may be responsible for the rather small portions of stimulus material remembered and should be investigated in more detail in the future to further support our interpretation of unaffected memory processes in BPD.

Analyzing attributional style revealed marked alterations in assigning causes to both positive and negative events in BPD that was linked particularly in the case of negative events to the negative evaluation bias for positive and neutral stimuli. Compared to healthy subjects, BPD patients attributed the cause for negative events in a more internal, global, and stable manner to themselves. Similarly, they explained positive events as less caused by themselves, as less stable in time and less global. Thus, our data are in line with the findings of Moritz et al. [21] in that BPD patients more often explain negative events as caused by themselves. Our data suggest that this is combined with a more global and stable attributional style in the sense that BPD patients assume they are responsible for negative events across different situations and that this will not change with time. However, our findings do not support the idea that BPD patients over-attribute causes to themselves in general, because positive events are less often explained as caused by the patients themselves compared to healthy controls. Additionally, positive events were assumed to happen by chance and restricted to certain situations. Our findings fit with the attributional style described in the past for patients suffering from depressive disorders. However, in the present study, the percentage of BPD patients with current depression was very low.

Our data links the attributional style along all three dimensions, i.e. internality, stability and globality, to alterations in self-referential processing during a valence judgment task in the BPD group. Particularly the attribution of negative events was linked differentially in both groups to self-referential processing: in BPD patients, a more negative evaluation of self- vs. other-related positive and neutral words was associated with a tendency to assign internal, stable, and global causes to negative events. Attributional style has been linked to social functioning in everyday life in the past [24–26,43]. Thus, in BPD, altered positive self-referential evaluations may be related to the way causes of events are perceived in everyday life as well as to social functioning [44]. One may speculate whether by targeting these more basal self-referential processes in psychotherapeutical interventions, a change of the attributional style of subjects may be achieved that in turn may improve social functioning in everyday life of the patients.

Some limitations of the present study have to be addressed. Data were collected from a female sample which restricts the possibility to generalize our findings to male BPD patients. Since a large portion (36.6%) of the BPD patients was treated with psychotropic substances, the contribution of medication and, more specifically, differential medication to our findings is unclear. Additional analyses taking the medication status as covariate into account revealed no effect of medication suggesting that our findings are not caused by the influence of psychopharmacological treatment. Nevertheless, different medications have been used and all of them may affect cognitive and social cognitive functioning (e.g. [45–47]). Lazarus, Cheavens, Festa, & Rosenthal [48]emphasized that the effect of different psychotropic substances may interact with symptoms of both BPD and co-morbid disorders, and may result in potentially opposite effects on performance in social cognitive tasks across subjects. In the present study, the sample size limits the possibility of disentangling the contributions of different psychopharmacological substances to our findings. Therefore, further studies are required that investigate how different psychopharmacological substances may affect self-referential processing in BPD. Moreover, since BPD patients constitute a highly heterogeneous sample, further studies are required to analyze whether the alterations can be linked to specific co-morbidities such as social anxiety or posttraumatic stress disorder. Crucially, we found no co-variation of the alterations in self-referenced processing with the severity of depressive symptoms in the BPD patients which might have been expected based on previous studies on negative bias in mood disorders (for a review see [49]). Furthermore, one may argue that the word stimuli used are less ecologically valid compared to pictures and movies as used in previous studies. However, words provide easily-controllable stimuli and allow for stimulus-driven variation in self-reference without using different instructions. In addition, words can be linked with concrete emotional stimuli [50,51] and in this way transferred to more complex stimuli with higher ecologic validity such as pictures or even movies.

In sum, our study provides an important first step in understanding the role of altered self-referential processing in BPD. Our findings revealed that BPD patients exhibit a specific negative evaluation bias for self-related positive and neutral information. This bias appeared also—although less pronounced− when no explicit reference context was provided. Therefore, our findings imply that BPD patients do not overemphasize the intensity of negative information, but instead de-evaluate positive or neutral information, specifically when it is related to the self. Moreover, this was linked to patients’ attributional style, indicating that this evaluation effects may also be associated with BPD patients’ interpretation and anticipation of everyday life situations, including social encounters. For example, the less positive a BPD patients thinks of herself, the more she will anticipate that negative experiences such as rejection are caused by herself and reoccur, even in different contexts. This may well affect and impair BPD patients’ social functioning [52].

From a methodological point of view, our findings imply that it is particularly important to include not only negative, but also positive stimuli in future studies on BPD because evaluation may be altered particularly when positive stimuli are examined. In addition, our results show that attention should be devoted to the explicitly or implicitly given referential context when studying evaluation processes in BPD. For example, in BPD evaluation of emotional stimuli is often assessed without any particular instruction, leaving it open to whether the stimulus should be referred to the self or another person. In the present study, this was experimentally well controlled and doing so shed new light on the evaluation bias in BPD.

Regarding BPD therapy, our findings emphasize the relevance of cognitive interventions that specifically target evaluation processes of self-related, positive, and neutral information, besides training evaluation processes in general or the processing of negative information. In BPD patients, this may help to improve self-esteem and emotional state and may have additional positive effects on social functioning, self-esteem, and emotion regulation [12,13]. Beyond that, our findings suggest that the alterations in self-referential processes generalize to the processing of information without an explicitly defined social context. This emphasizes the relevance of training discrimination abilities to counteract an overgeneralization to information without a referential context. To be able to develop specific therapeutical interventions, a deeper understanding of the affected sub-processes seems essential. Further studies extending this paradigm with help of brain imaging methods such as event related potentials and functional magnetic resonance imaging may allow for a mental chronometry of self-referential processing and the localization of BPD specific deficits in the time course of self-referential processing.
